# In-plane tunnelling field-effect transistor integrated on Silicon

**DOI:** 10.1038/srep14367

**Published:** 2015-09-25

**Authors:** Ignasi Fina, Geanina Apachitei, Daniele Preziosi, Hakan Deniz, Dominik Kriegner, Xavier Marti, Marin Alexe

**Affiliations:** 1 Max Planck Institute of Microstructure Physics, Weinberg 2, Halle (Saale), D-06120, Germany; 2 Department of Physics, University of Warwick, Coventry CV 4 7AL, United Kingdom; 3 ICN2-Institut Català de Nanociència i Nanotecnologia, Campus Universitat Autònoma de Barcelona, 08193 Bellaterra, Spain; 4 Department of Condensed Matter Physics, Faculty of Mathematics and, Physics, Charles University, CZ-121 16 Prague 2, Czech Republic; 5 Department of Spintronics and Nanoelectronics, Institute of Physics ASCR, v.v.i., Cukrovarnická 10, 162 53 Praha 6, Czech Republic

## Abstract

Silicon has persevered as the primary substrate of microelectronics during last decades. During last years, it has been gradually embracing the integration of ferroelectricity and ferromagnetism. The successful incorporation of these two functionalities to silicon has delivered the desired non-volatility via charge-effects and giant magneto-resistance. On the other hand, there has been a numerous demonstrations of the so-called magnetoelectric effect (coupling between ferroelectric and ferromagnetic order) using nearly-perfect heterostructures. However, the scrutiny of the ingredients that lead to magnetoelectric coupling, namely magnetic moment and a conducting channel, does not necessarily require structural perfection. In this work, we circumvent the stringent requirements for epilayers while preserving the magnetoelectric functionality in a silicon-integrated device. Additionally, we have identified an in-plane tunnelling mechanism which responds to a vertical electric field. This genuine electroresistance effect is distinct from known resistive-switching or tunnel electro resistance.

Electric modulation of the magnetic properties of a nanoscale ferromagnetic semiconductor (FM-SC) at room temperature, in a system integrated with Si is a challenge for the scientific community^1^. One of the most appealing device architectures to achieve this challenging objective is the ferroelectric field effect transistor (FeFET) where the channel is a FM-SC, which fulfils the major requirements of achieving both magnetic and transport modulation in a reversible way, while operating at modest voltages, low current, and very short time scale^2^. In a classical non-magnetic FeFET device the transport across the semiconductor channel is modulated by the charge accumulated at the ferroelectric gate using the same FET architecture^3^. In FeFET based on FM-SC, both the magnetic and transport properties are modulated by the depleted and accumulated charge. FeFET based on FM-SC is in fact a multiferroic device presenting ferroelectric and ferromagnetic order. In multiferroic composites (combining ferroelectric and ferromagnetic materials) strain mediated coupling between the electric and the magnetic order (magnetoelectric coupling) is commonly exploited^4^,^5^,^6^,^7^. However, in devices where coupling is mediated by strain, the mechanical clamping issue appears^8^, which would be avoided in a multiferroic FeFET, without requiring nanostructuring^9^,^10^,^11^.

On one hand, FeFET devices where the semiconductor channel is a FM-SC have been already realized^12^,^13^,^14^,^15^,^16^,^17^ with low ordering temperatures. On the other hand, in the field of oxide electronics the development and the study of FeFET devices, with a strongly correlated material (SCM)^18^ acting as channel, is still object of intensive research. Noticeably, it is the work on the rare earth manganites family, using the archetypical La_1−x_Sr_x_MnO_3_ as a channel material, which showed the largest modulation on the magnetic properties induced by ferroelectric polarization reversal^19^,^20^. The observed shift of the Curie temperature (T_c_) for the ferroelectrically-gated SCM is commonly much larger than the one measured for a FM-SC channel (compare, for instance, refs 15 and 19). Moreover, T_c_ values characterizing the SCMs are also larger than in FM-SCs. The latter fact is of great interest since a room temperature FM-SC has been predicted^21^, but never been realized, and SCM ferromagnetic at room temperature can be an alternative to them. The major drawback on the studies of SCMs is that the aforementioned effects have been observed only in high-quality epitaxial films, usually grown on top of expensive substrates such as SrTiO_3_ with vicinal surfaces, which make them non-suitable for any commercial application. Therefore, there is an important need to study similar magnetoelectric effects on less demanding systems grown on substrates, which are compatible with nowadays microelectronic or MEMS industry such as silicon.

Here we report on a La_0.825_Sr_0.175_MnO_3_/Pb_0.2_Zr_0.8_TiO_3_ (LSMO/PZT) bilayer directly grown on as-received Si substrate as sketched in Fig. 1a. The Sr content of LSMO has been selected to be equal to 0.175, exactly at the verge between metallic and insulating phases^22^, behaving in bulk more as a semiconducting oxide. We show that the modulation of the magnetic and transport properties of LSMO upon PZT ferroelectric switching is large, as revealed by a remarkable change in T_c_, as well as a change of absolute resistance and magnetoresistance, despite the polycrystalline nature of the structure. Our experimental data allow us to infer a possible distinct mechanism (not present on similar epitaxial systems) that accounts for the observed transport modulation. The results presented here show the advantages of the use of a SCM as a channel, with large effects and high order temperature, in a structure grown on Si.

## Results

**Figure 1:**
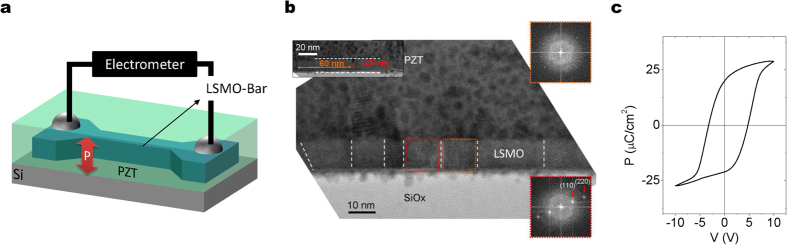
(**a**) Sketch of the field effect device and the contact configuration in the transport measurements. (**b**) Cross-sectional high resolution TEM image revealing the grainy morphology of PZT and LSMO and the polycrystalline nature of LSMO. Dashed white lines mark the grain boundary of the LSMO layer. FFT of the highlighted red and green regions are shown in the insets of the respective colours. (**c**) Ferroelectric P-V loop recorded for Si/LSMO/PZT.

Cross-sectional high-resolution Transmission Electron Microscopy (TEM) image is shown in Fig. 1b, in which the LSMO and PZT layers can be clearly distinguished. From the TEM image a grainy morphology for PZT is inferred. The LSMO layer exhibits grain sizes ranging from 5 to 30 nm (the grainy morphology is confirmed by the AFM characterization shown in Fig. S1 in Supplementary information). Several of these grains are crystalline, as revealed by the FFT (bottom inset) of the marked area (in red) and labelled using pseudo-cubic notation. Other grains show no peaks on the FFT (top inset), because of the non-proper orientation of the crystalline planes with respect to electron beam. Both facts point towards the polycrystalline nature of LSMO (as it is also the case for PZT), as further confirmed by the X-ray characterization (see Fig. S2 in Supplementary information).

As shown in Fig. 1c, the polycrystalline nature of the PZT layer has not hampered obtaining a sizeable remnant polarization (P_r_ ≈ 25 μC/cm^2^). The P_r_ value is comparable to that obtained for good crystalline ferroelectric films on buffered Si^23^. The visible shift towards positive voltages indicates polarization imprint with P pointing outwards LSMO.

**Figure 2:**
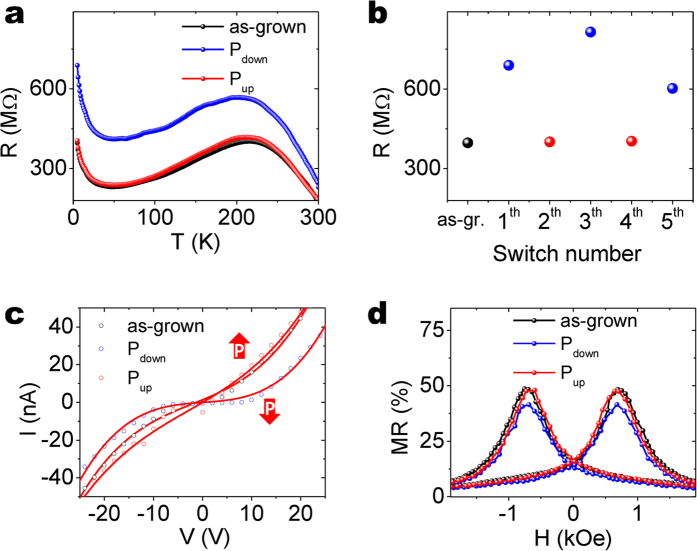
(**a**) Resistance dependence on temperature for as-grown, P_up_ and P_down_ polar states. (**b**) Resistance values measured at 5 K upon successive ferroelectric switching of the PZT layer. Data in (**a**) correspond to the as-grown and 1^th^ and 2^th^ states of (**b**). (**c**) I-V characteristics of the LSMO bar for the as-grown state, P_down_ and P_up_ at 5 K. (**d**) Magnetoresistance measurement with MR = (R(H) − R(50kOe))/R(50kOe) for the as-grown state, P_down_ and P_up_ at 5 K.

The influence of the polarization of the PZT layer on the electronic transport of the underneath LSMO layer is revealed by the dependence on temperature of the LSMO resistance acquired for three different polar states of PZT. In Fig. 2a, the black line, measured for the PZT as-grown state, shows three distinctive regions. At low and high temperature the slope is negative (semiconductor-like) whereas at intermediate temperatures it is positive (metallic-like). The minimum of the resistance at ≈50 K is the so-called up-turn temperature (T_up-turn_) and its origin is discussed in detail below. The distinctive peak at 216 K, at which LSMO undergoes an insulating to metallic transition (T_MI_, determined from the curve derivative) marks the onset of the ferromagnetic order^22^. Using a conductive AFM tip the ferroelectric polarization of PZT has been switched from the as-grown state to P_down_ (with +9 V), all along the LSMO bar. Complementary PFM characterization to crosscheck the polarization switch can be seen in Fig. S3 in Supplementary information. Afterwards, the resistance dependence on temperature (blue line in Fig. 2a) has been measured under the same conditions. Comparing the two curves before and after polarization switching, two main features can be observed: i) the resistance significantly increases, shifting the whole curve, at room temperature; the shift is 35% (in agreement with previous results in ref. 24) and it increases up to almost 75% at low temperature, and ii) the T_MI_ shifts from 216 (P_up_) to 200 (P_down_). After switching to P_up_ (−9 V) the measured R(T) curve overlaps to the one obtained in the as-grown state. The latter finding is expected, since P in the as-grown state is mainly P_up_ (Fig. S3 in Supplementary information). Upon successive ferroelectric switching results similar to those shown in Fig. 2a have been recorded, as it can be seen in Fig. 2b. As expected, the resistance states corresponding to P_down_ are more scattered because of the aforementioned presence of internal imprint field; the imprint produces some back-switching towards P_up_ (not controllable in our device architecture), that hinders the possibility to obtain the fully polarized P_down_ state. Note that the characterized bilayer grown on Si allows disregarding strain as the parameter that mediates the found magnetoelectric effect between PZT and LSMO because of the substrate clamping effect that forbids the LSMO lattice modulation.

We now focus on the transport and magnetotransport properties of LSMO at low temperatures, where LSMO behaves as semiconductor and it is ferromagnetic. The current versus voltage (I-V) characteristics recorded for the three polar states at 5 K are depicted in Fig. 2c. It can be readily observed that the switch of polarization state induces significant changes in the I-V characteristics especially in the low voltage range, where a remarkable shift of the onset is observed. Magnetotransport measurements for the three polar states have been also performed and they are shown in Fig. 2d. Significantly, the obtained value for the absolute magnetoresistance (MR = (R(H) − R(50kOe))/R(50kOe)) is large (near 50%), and the hysteresis with coercive field at 0.7 kOe is in agreement with the performed magnetic characterization, see Fig. S4 in Supplementary information. The small, but clearly visible difference in the amplitude of the magnetoresistance before and after ferroelectric switching is also worth noting. The mechanisms that account for the observed difference in the electrical resistance (I-V characteristics) and the magnetoresistance upon ferroelectric switching and their implications on the measured resistance change are discussed in the following section.

## Discussion

The remarkable change in the metal-insulator transition temperature T_MI_, and the change in the magnetoresistance amplitude upon ferroelectric switching univocally point to the fact that the magnetism is modulated by the field effect in the studied system. However, the peculiar crystalline character and the rather unexplored LSMO composition make the presented results not really comparable to those obtained in similar epitaxial structures. This raises the question whether the conduction mechanism at low temperature in this system is the same and how the different structural and morphological characteristics of our system can affect the obtained electric field effect as compared to the single-crystalline ones.

**Figure 3:**
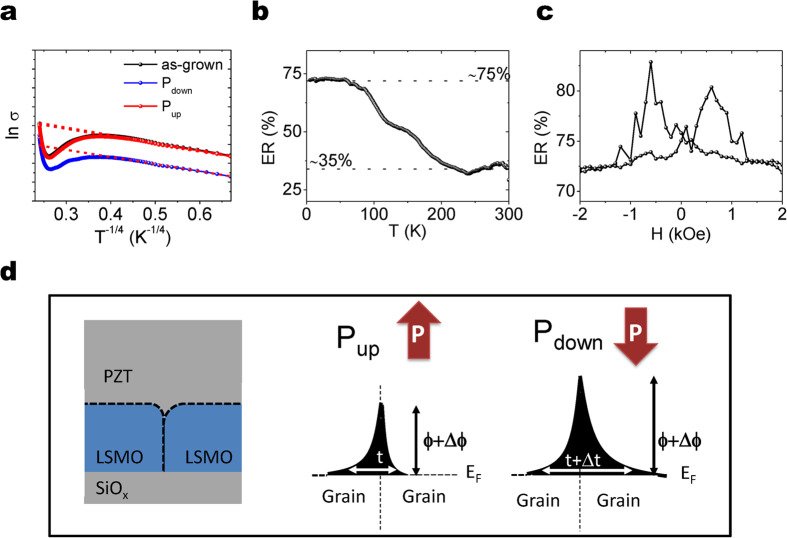
(**a**) l*n σ* versus *T*^−1/4^ plot for the as-grown state, P_down_ and P_up_. (**b**) Calculated electroresistance 
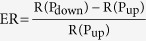
 as a function of temperature. (**c**) Electroresistance as a function of magnetic field measured at 5 K. (**d**) Upon ferroelectric polarization switching of PZT the grain boundary generated barrier changes its effective shape resulting in a change of the effective thickness of the tunnelling barrier and, concomitantly, in the observed change of resistance.

It has been already proposed that a phase coexistence of metallic and insulating regions at T < T_c_ can play an important role, explaining the high resistivity found at T < T_up-turn_ and the dR/dT change of the sign at T_up-turn_^25^. In fact, scanning tunnelling microscopy studies in LSMO grainy samples have revealed that less conductive regions at the grain boundaries coexist with more conductive ones in the grains^26^. It appears natural to propose variable range hopping (VRH, present in epitaxial films) to describe the conduction mechanism^27^. In Fig. 3a the lnσ vs. T^−1/4^ plot is shown. The data have been fitted to 
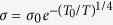
 that describes the VRH, where 
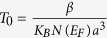
 and 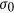 accounts for the conductance at infinite temperature, *N*(*E*_*F*_) for the density of states at the Fermi level, 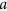 for the radius of the localized states, *β* is a constant, and *K*_*B*_ is the Boltzmann’s constant. T_0_ from the linear fit is extracted and a non-significant difference between the P_down_ (T_0_ = 13 K) and P_up_ (T_0_ = 16 K) states is obtained. Further analysis shows that these results are at odds with precedent works, where it was concluded that transport is modulated by a change in the number of carriers upon ferroelectric switching^19^. Under this scenario, P_down_ sets the electronic state of depletion at LSMO (accumulation for P_up_) with a subsequent decrease of charge carriers (transport in LSMO is governed by holes); therefore, T_0_ must be larger for P_down_ than for P_up_. In fact, in epitaxial bilayer films of the same LSMO as the one analysed here, the modulation takes place and it is remarkably large (see Fig. S5 in Supplementary information and ref. 28). This points to the fact that: even though VRH participates in the conduction process, the change of conductivity upon polarization switching is not dictated by variations of *N*(*E*_*F*_).

The failure to explain the obtained resistance modulation by VRH at low temperature suggests that another transport mechanism can play an important role. The particular influence of the grain boundaries on the conductivity in the (La_x_A_1−x_)MnO_3_ family has been exhaustively studied^29^ and it has been proposed that the conductivity at T < T_up-turn_ can be influenced by the tunnelling current between different grains; therefore grain boundaries act as an insulating layer^30^,^31^,^32^. A tunnelling barrier with several localized states in the insulator follows the relationship I = V·G(V), where G(V) = G_0_ + G_1_ + G_2_V^4/3^ + G_3_V^5/2^ after Glazman and Matveev^33^, and it can be used to describe the obtained transport properties in grainy (La_x_A_1−x_)MnO_3_ films as demonstrated in detail in ref. 30, and more recent works in ref. 34. In Fig. 2c, the data have been fitted by the aforementioned Glazman-Matveev equation (the goodness of the fit and the fitted parameters are presented in Table S6 in Supplementary information). The good quality of the fitting, in agreement with the resemblance of the I-V characteristics shown in ref. 30 for samples where the tunnelling through grains is predominant, indicates that for P_down_ the contribution of the tunnelling current is smaller than for P_up_. Interestingly, in the ohmic regime (low V), where G(V) ≈ G_0_ + G_1_, the tunnelling resistivity shows a large change from ≈3 (for P_up_) to ≈27 (for P_down_) kΩ·cm (near 1000%).

Tunnelling is a temperature independent effect; therefore the found modulation must be temperature independent. This is confirmed by the dependence of the electro-resistance 
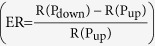
 on temperature shown in Fig. 3b that displays a plateau at temperatures below T_up-turn_. Note that the term “electroresistance” does not have the same physical origin as resistive-switching, or tunnel electroresistance based devices. Here it accounts for the modulation of the tunnel magnetoresistance between LSMO grains by electric field. When the temperature is further increased above T_up-turn_, the electroresistance is no longer limited by tunnelling, which results in a gradual decrease of ER. This is in agreement with the quantum-mechanical origin of the tunnelling current, and with the coexistence of tunnelling and overbarrier current (not tunnelling) as already introduced in ref. 35. The ER plateau not found until 200 K indicates that tunnelling is still present at this very high temperature. In Fig. 3c, it is shown the dependence of the electroresistance on magnetic field. It can be inferred that it reaches a maximum near the magnetic coercive field, indicating that the spin-polarization (which depends on the particular shape of the DOS) changes upon ferroelectric switching, which accounts for the modulation of magnetoresistance.

These results indicate that the observed change is dictated by the variation of effective shape of the tunnelling barrier between grains, instead of by the number of available carriers or localized states upon switching. To sum up, in Fig. 3d, we have sketched the present scenario. In Fig. 3d (left panel), it is depicted a grain boundary in between two LSMO grains (labelled). For P_up_ (Fig. 3d (middle panel)), the current will flow through tunnelling between different LSMO grains. The tunnel barrier shape is defined by its thickness and height, and the tunnelling probability decreases when both thickness and/or height increase. For P_down_ (Fig. 3d (right panel)), the grain boundary would change its properties, and therefore the effective thickness and height of the tunnel barrier are modified. According to the results presented here for P_down_ the tunnelling probability is less, which has been depicted in Fig. 3d by an increase of the tunnelling thickness and/or height. A plausible scenario is that the insulating grain boundaries^26^ can change their properties by the change in the electrical doping induced by the electric field. This results in a change in the effective insulating thickness, which accounts for a change in the tunnelling probability. Elucidating the particular origin (change in height or in thickness) of the modulation of the tunnelling current is beyond the scope of the present work.

To summarise, the shifting of T_MI_ by 16 K and the change of magnetoresistance upon ferroelectric switching are a clear indications of a large modification of the magnetic properties by an electric field. Remarkably, the T_MI_ shift of 16 K largely exceeds that of any other field-effect device based on FM-SC^15^, and it is similar to that found in similar epitaxial heterostructures^19^,^28^,^36^. The analysis of the obtained data allows us to conclude that the change of magnetic properties and the variations of the transport properties of the grain boundary (acting as a tunnelling barrier between grains) account for the observed resistance modulation upon ferroelectric switching. The dependence of ER on temperature indicates that the tunnelling current contribution is still present at least up to ~225 K. These results obtained in an integrated Si structure are of great interest for industrial applications. Moreover, the findings presented here claim for more investigation on horizontal tunnelling barriers (artificial or self-assembled and magnetic or electric) modulated by external electric field effect.

## Methods

### Sample growth

PZT/LSMO bilayers were fabricated by Pulsed Laser Deposition (PLD) on commercial as-received Si(001) wafers in a two steps process, following the same procedure described elsewhere^28^. Thickness of the samples has been determined by TEM measurements show in Fig. S7 of Supplementary information.

### TEM characterization

TEM investigation has been performed by using FEI TITAN 80–300 operated at 300 kV.

### Ferroelectric characterization

Macroscopic ferroelectric characterization was performed by measuring the dynamic P-V hysteresis loops at 10 Hz using a TFAnalyzer 2000 (aixACCT Systems, Aachen, Germany) using 60 × 60 μm^2^ Cu top electrodes *ex-situ* evaporated.

### Magnetotransport characterization

Transport measurements have been performed by using an electrometer (6517B Keithley Co.). The resistance of LSMO is measured using a two points contact method at 50 V (as sketched in Fig. 1a). Because of the large resistance of the LSMO thin film it can be assumed that the contact resistance does not largely contribute to the total measured resistance and a standard four-points measurements is not required. The fact of covering the LSMO with the PZT layer does not produce any significant effect on the LSMO transport and magnetotransport properties, disregarding the presence of any significant chemical change upon PZT deposition on LSMO, as can be inferred from transport measurement on LSMO sample before PZT deposition (Fig. S8 of Supplementary information). The sample was introduced into a PPMS device using a special designed insert (Quantum Design Co.) to allow the control of the magnetic field and the temperature. The used temperature rate was fixed to 2 K/min.

## Additional Information

**How to cite this article**: Fina, I. *et al.* In-plane tunnelling field-effect transistor integrated on Silicon. *Sci. Rep.* **5**, 14367; doi: 10.1038/srep14367 (2015).

## Supplementary Material

Supplementary information
