# Supplementary material for: In-plane tunnelling field-effect transistor integrated on Silicon
Source: Sci Rep. 2015 Sep 25;5:14367. doi: 10.1038/srep14367 (PMC4585907; doi:10.1038/srep14367)
Supplement: Supplementary information [file srep14367-s1.pdf]

Supplementary Information

In-plane tunnelling field-effect transistor integrated on Silicon

Ignasi Fina, Geanina Apachitei, Daniele Preziosi, Hakan Deniz, Dominik Kriegner, Xavier Marti, Marin Alexe

Supplementary information S1

The surface morphology of the samples was investigated using a Digital Instrument D-5000 atomic force microscope AFM. In figure S1, the AFM scan on the LSMO layer before the deposition of the top PZT layer shows a grainy morphology with sizes <50 nm.

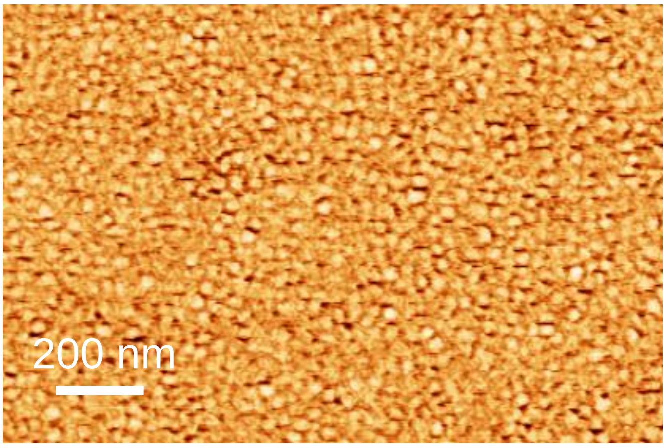

Figure S1. Deflection AFM image of the LSMO film before PZT deposition.

Supplementary information *S2*

X-ray diffraction (XRD) analyses were carried out using a Siemens D500 diffractometer with Cu-Kalpha radiation in Bragg-Brentano geometry. In order to avoid the strong diffraction from the single crystalline Si substrate and increase the contribution of the thin films we intentionally tilted the sample leading to the observation of only the diffuse scattering around the Si (004) Bragg diffraction position. The signal of the bilayer is not affected by aforementioned tilt due to its polycrystalline nature.). High Resolution

The X-ray diffraction (XRD) pattern (figure S2) of our bilayer shows several diffraction peaks due to the polycrystalline nature of the films. These correspond to the PZT layer (with lattice parameters a=3.95(1) Å and c=4.08(1) Å, P4mm notation, close to the bulk ones) and except one corresponding to the single crystalline Si substrate.

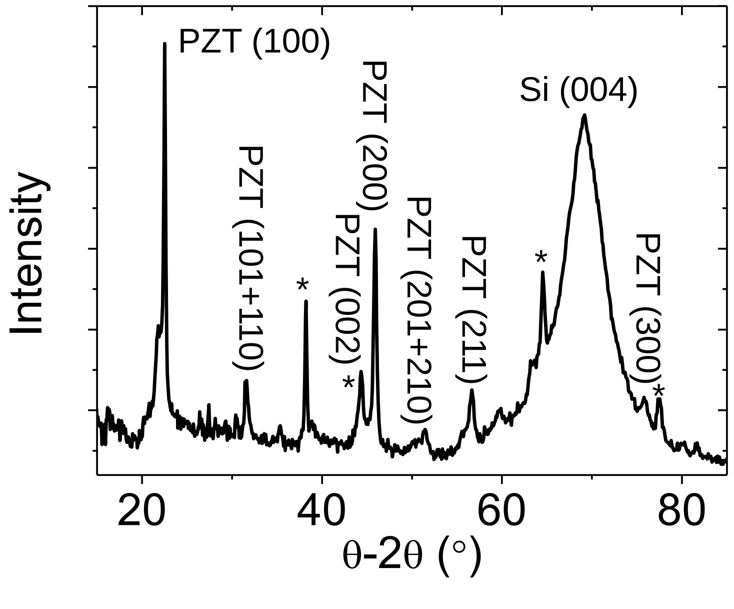

Figure S2. θ-2θ scan of Si/LSMO/PZT. By "*" are marked Ag-traces corresponding to residual silver used for contacting.

Supplementary information S3

Piezoelectric force microscopy (PFM) (XE100 Park system) was used to investigate the ferroelectric properties and the domain patterns of the sample.

180° piezo-phase contrast in the PFM image has been observed as depicted in figures S3a,b. Bright and dark regions of the image are indicative of different regions, where either positive (+9 V, corresponding to a ferroelectric polarization pointing towards LSMO, P_{down}) or negative (-9 V, ferroelectric polarization pointing outwards LSMO, P_{up}) voltage bias has been applied, respectively. The outmost region in dark reveals that the as-grown PZT layer shows an imprinted polarization pointing outwards LSMO. As expected, in the poled regions, the piezo-amplitude image (figure S3a) shows no-contrast except at the domain walls. Note also the noticeable roughness of the domain walls between domains of different polarization; this is the consequence of the grainy morphology of the sample, which in any case does not hamper the switching of large ferroelectric domains.

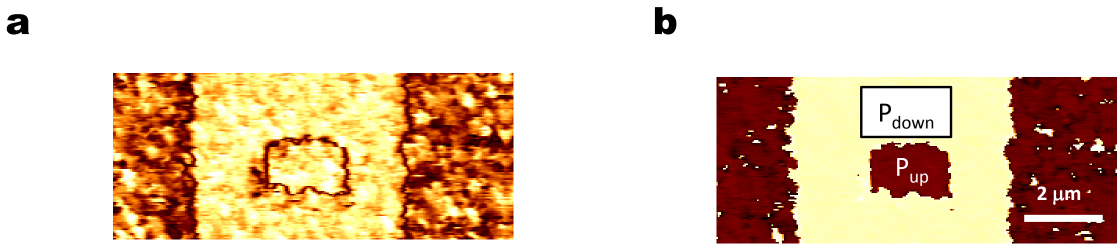

Figure S3. (a) PFM-amplitude and (b) PFM-phase images of Si/LSMO/PZT. The PFM-amplitude is in arbitrary units. The contrast in (b) is 180°.

Supplementary information S4

The magnetic characterization was performed with a Quantum Design SQUID magnetometer. Raw measurements of the magnetization vs applied magnetic field were corrected for the contributions from the substrate by substracting the linear response measured at high fields.

Figure S4, shows the M(H) loop recorded for the LSMO/PZT bilayer at 5K.

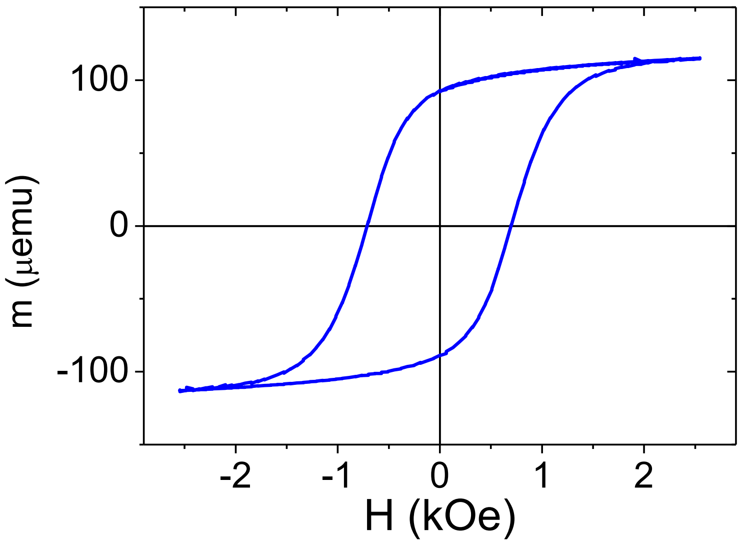

Figure S4. M(H) loop recorded at 5K

Supplementary information S5

Figure S5 shows the temperature dependence of the conductivity in the Arrhenius $T^{-1/4}$ representation for an epitaxial $SrTiO_3$(001)/LSMO/PZT sample. Further details on sample growth along with structural and morphological characterization can be found elsewhere[s1]. As a result, from the linear extrapolation of both σ(T) curves at low temperature, T_0 values of $6.2x10^6$ and $1.8x10^6$ K, were obtained for P_{down} and P_{up} states, respectively. Interestingly, the ratio between the T_0 values calculated for P_{down} and P_{up} states is near 4, much larger than the one calculated for the investigated sample in the main manuscript. Additionally (assuming that the localization length *a* for each states remains constant), the scenario in which the transport is dictated by a modulation of the number of carriers upon ferroelectric switching is in agreement with the trend of the aforementioned ratio value. Indeed, P_{down} sets the electronic state of depletion with a subsequent decrease of charge carriers (transport in LSMO is dictated by holes), it is clear that T_0 must be larger for this electronic state ($T_0 \propto 1/N(E_F)$).

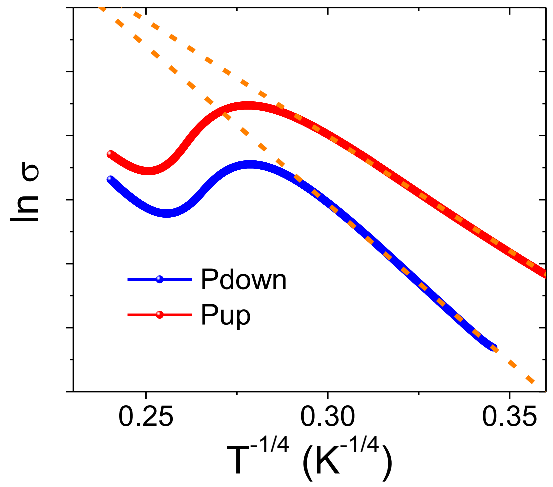

Figure S5. lnσ versus $T^{-1/4}$ plot for the as-grown state, P_{down} and P_{up} for epitaxial epitaxial $SrTiO_3$(001)/LSMO/PZT bilayer.

Supplementary information S6

In table S6, the results of the fittings according to the Glazrnan-Matveev equation for the three ferrolectric polar states are summarized. The remarkably smaller G_0+G_1 and G_2 for Pdown indicate much less probability of tunnelling across the averaged barrier. Instead, G_3 is constant for the three evaluated polar states. Remarkable, is the fact that for P_{down} the χ^2 is smaller, indicating that the data is better described by the equation and that the tunnelling is more important.

Table S6. Summary of the fitting parameters obtained using the equation I = V·G(V), where $G(V)= G_0+G_1+G_2V^{4/3}+G_3V^{5/2}$ is fitted to the data of the figure 2c.

	G_0+G_1 (pS)	G_2 ($pS\cdot V^{-4/3}$)	G_3 ($pS\cdot V^{-5/2}$)	$\chi 2$
$P_{as\text{-}grown}$	1130(5)	13(6)	1,8(3)	6,6 $x10^{-18}$
P_{down}	180(1)	0,5(6)	2,3(3)	2,3 $x10^{-18}$
P_{up}	1526(2)	12(8)	1,4(2)	7,4 $x10^{-18}$

Supplementary informationS7

In figure S7, it is shown the cross-section used to determine the layers thickness.

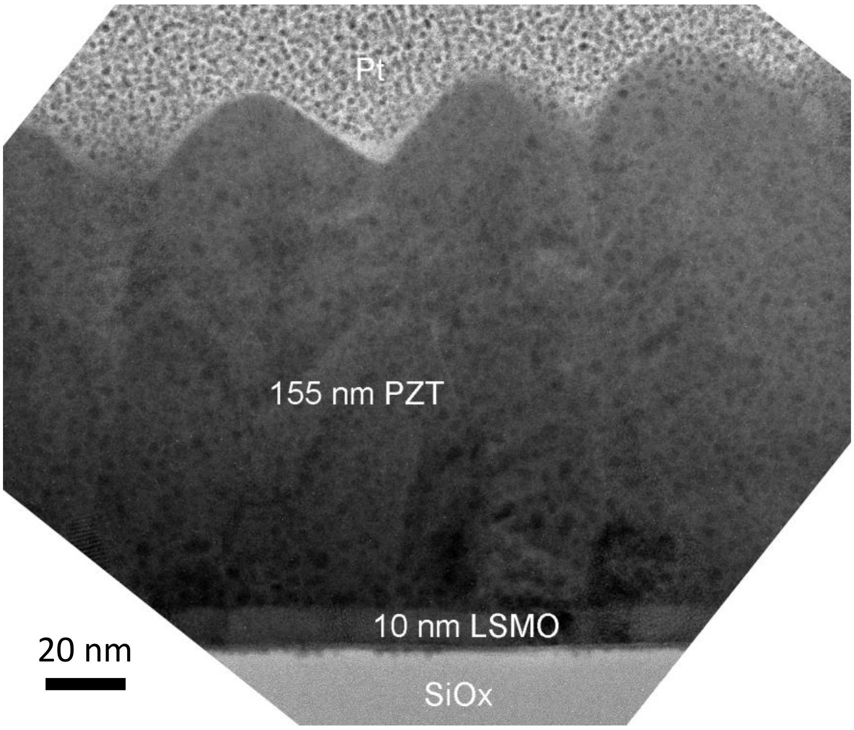

Figure S7. Cross sectional high resolution TEM image of Si/LSMO/PZT sample.

Supplementary information S8

Transport measurements performed on the LSMO layer before PZT deposition shown in figure S8 show similar values of resistance for LSMO compared with figure 2a indicating no chemical reaction between PZT and LSMO.

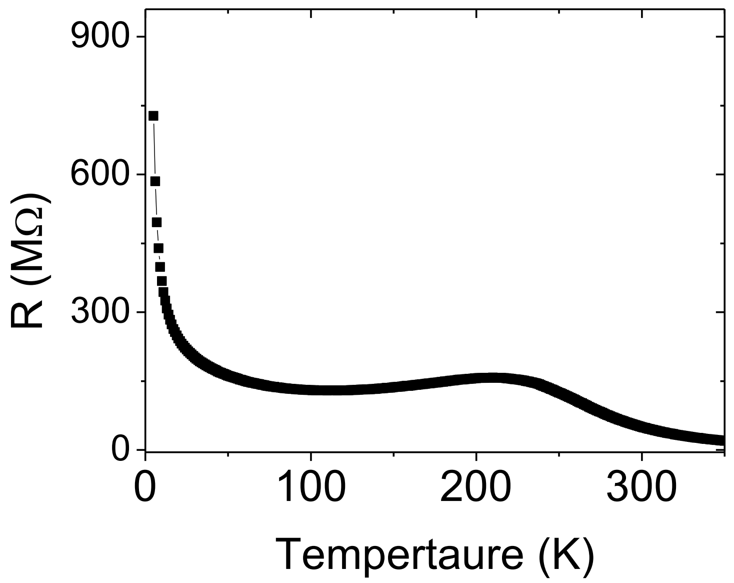

Figure S8. Resistance dependence on temperature of the bare LSMO layer (before PZT deposition) at various fields.
